# Advances of Proteomic Sciences in Dentistry

**DOI:** 10.3390/ijms17050728

**Published:** 2016-05-13

**Authors:** Zohaib Khurshid, Sana Zohaib, Shariq Najeeb, Muhammad Sohail Zafar, Rabia Rehman, Ihtesham Ur Rehman

**Affiliations:** 1 Department of Prosthodontics and Dental Materials, School of Dentistry, King Faisal University, Al-Hofuf 31982, Saudi Arabia; drzohaibkhurshid@gmail.com; 2 Department of Biomedical Engineering, College of Engineering, King Faisal University, Al-Hofuf 31982, Saudi Arabia; szohaib@kfu.edu.sa (S.Z.); rimtiaz@kfu.edu.sa (R.R.); 3 Department of Restorative Dental Sciences, Al Farabi Colleges, Riyadh 11313, Saudi Arabia; shariqnajeeb@gmail.com; 4 Department of Restorative Dentistry, College of Dentistry, Taibah University, Madina Munawwarrah 41311, Saudi Arabia; MZAFAR@taibahu.edu.sa; 5 Department of Materials Science and Engineering, The Kroto Research Institute, The University of Sheffield, North Campus, Broad Lane, Sheffield S3 7HQ, UK

**Keywords:** proteomics, dentistry, enamel, dentin, saliva, gingival crevicular fluids and dental materials

## Abstract

Applications of proteomics tools revolutionized various biomedical disciplines such as genetics, molecular biology, medicine, and dentistry. The aim of this review is to highlight the major milestones in proteomics in dentistry during the last fifteen years. Human oral cavity contains hard and soft tissues and various biofluids including saliva and crevicular fluid. Proteomics has brought revolution in dentistry by helping in the early diagnosis of various diseases identified by the detection of numerous biomarkers present in the oral fluids. This paper covers the role of proteomics tools for the analysis of oral tissues. In addition, dental materials proteomics and their future directions are discussed.

## 1. Introduction

Every living thing contains fascinating molecules called proteins [1]. Proteins are building blocks for the living matrix and perform various functions [2]. The human body contains a number of different proteins which are structural, catalytic, regulatory, transport and storage, and transducer proteins. Each of these proteins plays a specific functional role [3]. Proteins belong to biological macromolecules that exist as three-dimensional structures because of the sequences involving the twenty different amino acids [4,5]. These amino acids are linked with each other by peptide bonds. The word “proteome”, first coined by Mark Wilkins in 1961, is used to describe a mixture of proteins [6]. All proteomes arise from mRNA and can be used to describe a cell’s protein content [7]. In simple terms, proteomics is the study of the distribution and interaction of proteins in time and space in a cell, organisms, or an ecosystem. In recent years, a number of proteomical studies on human body fluid and tissues (diseased and non-diseased) have been carried out by several researchers to analyze the chemistry in order to understand the life processes at the molecular as well as the cellular levels [8,9].

Proteomic tools have the ability to analyze human body samples such as blood, saliva, serums, urine, cervico-vaginal fluid (CVF), sperm cells, gingival crevicular fluids (GCF), microorganisms, and different tissues (enamel, dentine, cementum, pulp, gingiva, bone ligaments, stem cells, and mucosa) in both pathological and normal physiological states [10,11,12]. Quite a few studies have reported the analysis of dental tissues by means of proteomic tools. Approximately, 64% of human oral tissues samples have been studied for proteomics analysis as compared to 11% of animal dental tissues which signifies the clinical importance of proteomics [13]. A number of studies have explored human saliva due to its ease of accessibility and non-invasive method of collection. A significant number of studies (30%) have explored the salivary proteome during oral pathological conditions e.g., caries, periodontitis, gingivitis, dental abscess, endodontic lesions, and oral carcinomas [14]. Two methods exist to conduct proteomics of body fluids. In top down proteomics, intact proteins are analyzed by Electrospray Ionization (ESI) or Matrix-Assisted Laser Desorption/Ionization (MALDI) and the peptides are generated by a gas phase fragmentation method [15]. In contrast, bottom-up approach of proteomics is commonly used to analyze peptides produced through chemical or enzymatic cleavage of proteins, and with post-translational modification as well as through liquid chromatography (LC) in conjunction with mass spectrometry (MS). The bottom-up analysis, the more conventional method, has been sometimes also called “shotgun proteomics” [16]. Both approaches are commonly used in proteomics procedures utilizing mass spectrometry.

Table 1 presents a description of proteomics studies carried out on whole mouth saliva (WS), unstimulated whole mouth saliva (USWS) parotid gland secretions, submandibular and sublingual gland secretions, salivary gland ductal secretions, enamel, dentine, pulp tissues, gingival crevicular fluid (GCF), cementum, alveolar bone, periodontal fibers (PDL), and dental materials through top-down and bottom up approaches.

Recent developments in dental proteomic have helped uncover previously unknown details regarding the unique protein structures and their function for the diagnosis, defense mechanisms, and regeneration of dental tissues, tissue calcification, and repairing of dental tissues [40]. The aim of this paper is to elaborate on the currently available techniques, their reported applications for dental tissues. Furthermore, the current status of dental proteomical analysis and the discovered biomarkers is discussed in detail.

## 2. Dental Hard Tissue Proteomics

The tooth is the strongest calcified tissue of the human body due to its special architecture and compositions. It is composed of three distinct mineralized hard tissues: enamel, dentine, and cementum. Enamel is the hardest tissue of the human body and contains 96% minerals, 1% proteins and the remainder being water. The adequate mechanical properties of enamel suit its primary function: mastication of food. Enamel, the only dental hard tissue formed before eruption of teeth, is formed by cells called ameloblasts. Histologically, the inorganic component of enamel is composed of micro-rods and inter-rods of hydroxyapatite (HA) crystals embedded in protein matrix, the organic phase [41]. To date, the major enamel proteins that have been recognized are amelogenin, ameloblastin, enamelin, and tuftelin [42]. Additionally, a total of 42 proteins has been identified during enamel formation (secretory phase and maturation phase) by two dimensional electrophoresis (2-DE) and MS. These proteins include ERp29 which is involved in secretory protein synthesis and calcium binding protein (calbindin) and play a role in tooth maturation [43,44,45,46]. It has been concluded that amelogenin takes part in enamel formation and cementum development by guiding cells. It also regulates initiation and growth of HA crystals during the mineralization front across the carboxyl terminals [47,48]. Very recently, a novel organic protein containing enamel matrix was reported in an adult human tooth with thickness of 100–400 µm which could provide important protein transportation or biochemical linkage between enamel and dentin [49]. Ameloblasts secrete enamel specific extracellular matrix protein called ameloblastin and its expression is also detected during the initial development of craniofacial bones and dental hard tissues of mesenchymal origin [50]. The precise role of ameloblastin is not known but it has been hypothesized that it may control the enamel mineralization process during tooth development alongside growth of enamel mineral crystals [51].

The bulk structure of a tooth is made from dentin which possesses neurogenic and regenerative capabilities. By weight, dentin contains 70% minerals (mainly hydroxyapatite), 20% organic component, and 10% water. In proteomics, dentin has been particularly found useful for the identification of collagenous and non-collagenous proteins [52]. Its formation and biomineralization (dentinogenesis) is dynamically complex. Odontoblasts develop and secrete extracellular matrix followed by mineralization in an organized fashion [53]. Most abundant collagenous proteins present in dentin matrix are collagen (type I, III, V, VI, and XII) providing a three dimensional (3D) template for the mineralization of apatite crystals. Fibronectin and matrix metalloproteinase (MMP) 2, 9, and 20 are associated with predentin collagen fibrils [36]. Park *et al.* [36] performed Sodium-Dodecyl-Sulfate-Polyacrylamide Gel Electrophoresis (SDS-PAGE) followed by an LC-MS/MS method for identifying dentin proteins. The outcome of these experiments revealed the presence of 233 proteins and was confirmed using Western blot technique and immunohistochemical staining. This study was the first to provide dentin protein classification such as: metabolic enzymes, signal transduction, cellular organization, transport, immune response, transcription factor activity, cell growth/maintenance, chaperone/stress response, nucleic acid binding, and unknowns function. Another study reported by Jagr *et al.* [54], 2-DE and nano-LC-MS/MS was used to identify 289 proteins overall of which 90 had been previously unknown. In this study nine novel proteins were identified and were classified as immunoglobulins which help in the formation of extracellular matrix, formation of the cytoskeleton, cell adhesion molecule activity, cytoskeleton protein binding, immune responses, and peptidase activity. These findings may provide deep insight for the regenerative and rehabilitation of dental tissues. Moreover, only a few studies reported the proteomics analysis of cementum and alveolar bone. A total of 235 and 213 proteins have been recognized in the alveolar bone and cementum respectively using LC-MS/MS with LTQ-FT (Ultra) due to their high resolution and high accuracy [33]. Previously, proteins including osteocalcin (BGLAP), TNN, FN, VIM, CHAD, vitronectin VTN, and LUM were identified as non-collagenous extracellular proteins in cementum and alveolar bone [55,56,57].

## 3. Oral Fluid Proteomics

**Figure 1:**
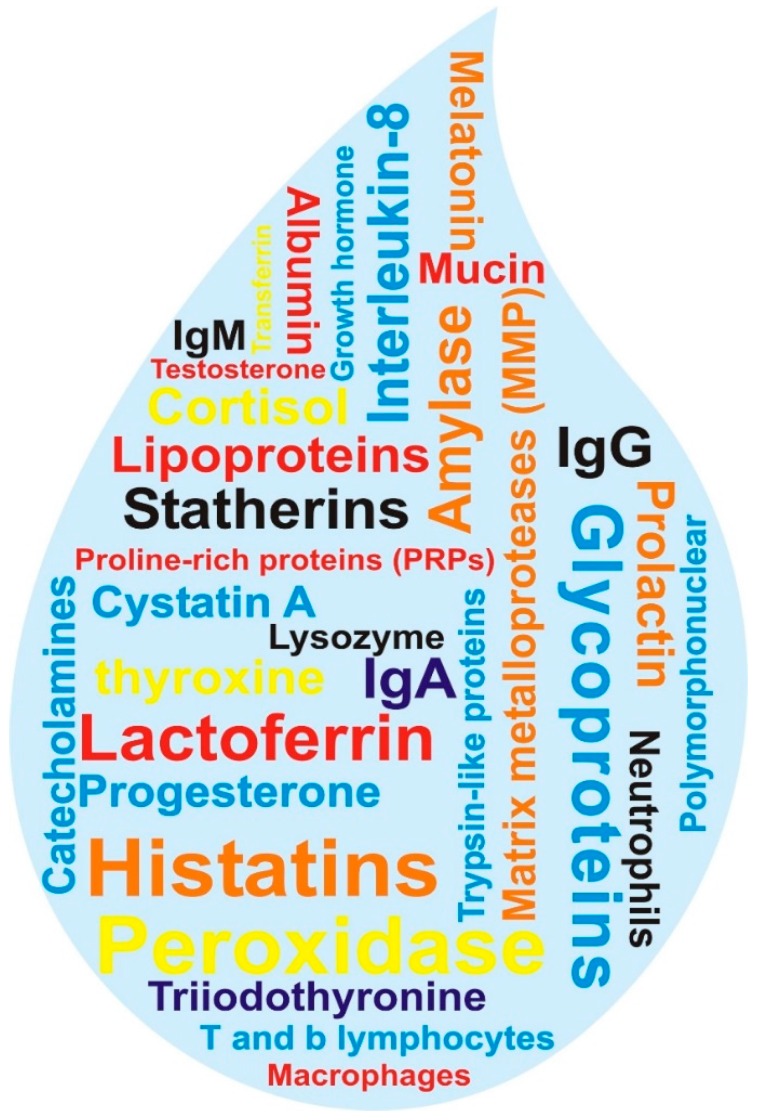
Illustration representing human salivary drop proteins and peptides.

Compared to dental hard tissues, whole mouth saliva (WMS) and GCF have been studied more for proteomical analysis due to their non-invasive collection technique, minimal patient discomfort and anxiety as compare to blood collection for serum or plasma [14]. WMS is not only composed of major and minor salivary glands secretions but also contains mucosal transudates from all surfaces of the mouth, lymphoid tissues, oropharynx, and GCFs [58]. Proteomics studies on human saliva revealed 1000 plus proteins and peptides (Figure 1).

Numerous studies have been conducted on WMS to evaluate various body physiological and pathological conditions and have proven it as a diagnostic as well as a maintenance test fluid. The WMS was isolated from different diseases such as dental caries, Sjögren’s syndrome, diabetic patients, breast cancer patients, squamous cell carcinoma patients, and graft-versus-host disease patients. The WMS has been analyzed successfully by proteomical tools (electrophorically and chromatographically) [59,60,61,62].

**Figure 2:**
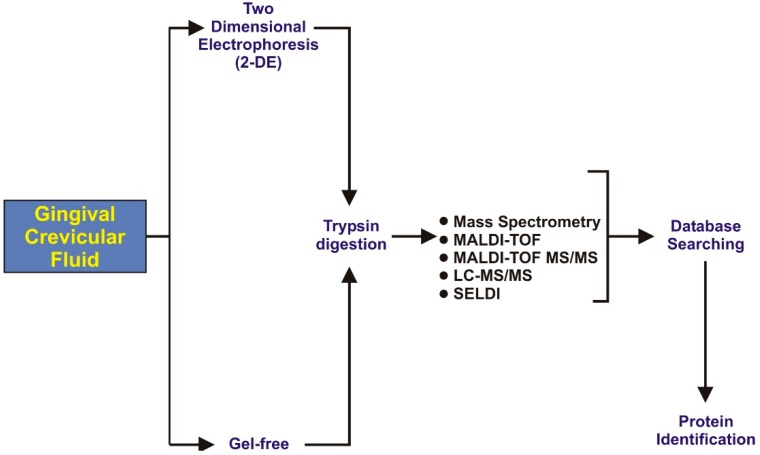
Illustration representing the steps of gingival crevicular fluids (GCF) proteomics analysis.

Human gingival crevicular fluid (GCF) has been analyzed extensively. GCF has a variable protein composition based on periodontal health and diseases. GCF contains serum transudate (found in gingival sulcus), broken products of host epithelial or connective tissues, subgingival microbial plaque, extracellular proteins, host inflammatory mediators and cells [63]. GCF provides medium for the transportation of bacterial byproducts into the periodontal microenvironment and also helps to drive off host derived products [64]. It has been reported that GCF volume for biochemical and proteomics analysis is limited due to severity of tissue inflammation [65]. Many methods are available for the collection of GCF such as paper strips, capillary tubes, gingival wash, and paper cones [63]. In the last decade researchers have favored using paper strip in their research work due to easy insertion into the gingival crevice up to 1 mm of depth without bleeding from periodontal pockets [35]. After collection of the GCF sample it goes through different steps for proteomics analysis, as illustrated in Figure 2.

Variety of proteolytic enzymes are identified in GCF, such as collagenase, elastase, and cathepsin B, D, H, and L [66]. These proteolytic enzymes have been reported as destructors of periodontal tissues and have the capability to degrade type-I collagen and glycoproteins [67]. Table 2 describes detailed profiling of GCF proteins, proteomic tools used, and the number of proteins identified. Most commonly reported identified proteins from GCF are actin, keratins, histones, annexins, proteins S100-A9, apolipoprotein A-1, albumin, salivary gland antimicrobial peptides (histatins, HNP-1, -2 & -3, LL-37, statherin), and cystatin B [68,69]. Some immune related proteins present in GCF such as; Ig γ-1 chain C region, Ig γ-3 chain C region, lactoferroxin-C, leukocyte elastase inhibitor, α 1 antitrypsin, heat shock protein β-1, and coronin-1A [70].

**Figure 3:**
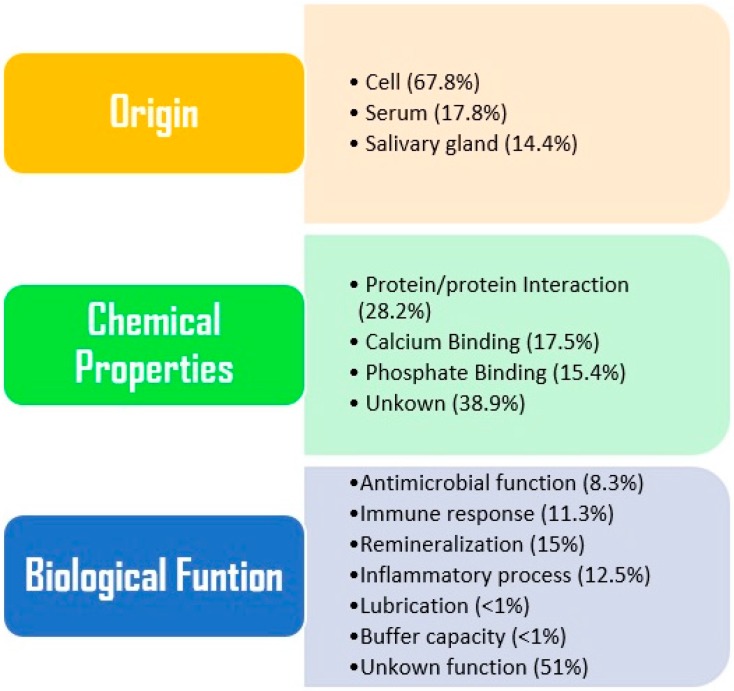
Classification of acquired enamel pellicle (AEP) proteins according to their origin, chemical function, and by their biological functions proposed by Siqueira *et al.* [84].

A protein based oral biofilm, the acquired enamel pellicle (AEP), is formed on tooth surfaces within seconds after mechanical cleaning of the tooth surfaces [75]. It consists predominantly of proteins secreted from major and minor salivary glands, carbohydrates, ions, exogenous proteins, and lipids [76]. Lee and co-workers investigated AEP layer on enamel and quantified 50 proteins through Liquid Chromatography- Electrospray Ionization-Mass Spectrometry(LC-ESI-MS/MS) [77]. This layer amount is approximately 0.5–1 µg per tooth surface and its formation is crucial and the dynamic process is influenced by many factors such as; circadian cycle, biochemical properties of tooth surfaces, proteolytic capacity of the oral micro environment, and the oral microbiota [78]. The fatty acids (FAs) identified in AEP play an essential role in the pellicle formation, bacterial adhesion and protection against pellicle [79]. *In-situ* study reveals qualitatively and quantitatively a wide range of FAs (C_12_–C_24_) through gas-chromatography- electrospray ionization/ mass spectrometry (GC-EI/MS), in this study pellicle were formed *in-situ* on bovine enamel slabs mounted on upper jaw splints and inserted in the mouth of 10 subjects for 3–240 min. Several methods have been used for the collection of AEP over the last four decades e.g., palatal appliances, chemical solubilization techniques, mechanical techniques and soaked membranes method [80]. All these methods reported different compositions due to different routes of collection. Mayhall *et al.* [81] remounted freshly extracted discs of teeth crowns in a palatal appliance worn by the subject for 1 h. After AEP formation on the specimens, they dipped the appliances in 2% HCl and detected glutamic acid, serine, and glycine but a low amount of proline [81]. In another *in vivo* study on AEP composition it revealed a high level of glutamic acid and alanine but a significant amount of hexosamines. This study also determined that a different approach of AEP collection varies the composition of AEP [82]. AEP has many function in the oral cavity such as lubrication, regulation of mineral homeostasis, providing defense against microbes and microbial colonization through specific receptors. Siqueira *et al.* [75] identified 100 plus proteins and peptides from *in vivo* AEP, and suggested that all play an active role in maintaining oral health. Similarly, histatin peptide has shown protective mechanism against demineralization of the tooth [83]. A total of 130 proteins were identified from AEP using LC-ESI-MS/MS with high confidence which allowed the classification of AEP proteins according to nature of origin, chemical properties, and biological function as shown in Figure 3 [39]. Very recently, another group of researchers has identified 76 proteins from *in vivo* AEP present on deciduous teeth through mass spectrometry which opens up a diagnostic frontier in pediatric dentistry [84].

## 4. Dental Soft Tissue Proteomics

This dental pulp is a soft connective tissue that is composed of cells (mesenchymal, odontoblasts, fibroblasts) neural fibers, blood vessels, and lymphatics [85]. Tooth development, nourishment, sensitivity, defense reactions, repair, and regeneration are the main functions of dental pulp [86]. Its unique composition helps in nutrition as well as sensation for external stimuli [87]. Robertson *et al.* [88] investigated the calcification response from dental pulp against various external stimuli including dental trauma, caries, abrasion or attrition, and tooth retransplantation. Similarly, Yamazoe *et al.* [89] harvested dental pulp cells in subcutaneous tissues and analyzed its calcified tissues through proteomics. The reason behind this is that stem cells have the potential to form deciduous or permanent pulp cells. In the last decade, dental pulp stem cells have proven value in repairing dentin-pulp complex [90]. Dental pulp contains unique tissue specific proteins and small leucine-rich proteoglycans (biglycan, lumican, and mimecan) [91]. Pääkkönen *et al.* [92] analyzed gene and protein expression in healthy and carious dental pulp organs for the first time using cDNA microarray and 2D-gel electrophoresis. In their study, slight expression changes were reported due to the high amount of healthy pulp in both conditions. In addition, a total of 96 proteins were identified through 2-DE gel followed by MS/MS techniques. In the same experiment, cDNA microarrays explored the difference of gene expression in carious tissues and no gene differences were detected in 96 detected proteins. In another *in vitro* study on proteome mapping of odontoblasts-like dental pulp revealed 23 total proteins by 2-DE gel followed by MS [93]. These proteins are comprised of various types of peptides such as cell membrane bound molecules, cytoskeleton, and nuclear proteins and are involved in matrix synthesis and enzyme metabolism. The expression of various recognized proteins (annexin VI, heteronuclear ribonuclear proteins C, collagen type VI, matrilin-2) were confirmed using western blotting (WB) technique and real time- polymerase chain reaction (RT-PCR) analysis. The RNA amplification technique was successfully used to analyze gene expression and protein encoding linked to physiology of dental pulp. Microarray analysis disclosed a total of 362 genes related to pulp expression specifically hence, further classified as protoncogenes, tooth morphogenesis, genes of collagen, DNAse, metallopeptidases, and growth factors [94]. McLachlan *et al.* [95] studied dental pulp tissues for detailed characterization and molecular changes due to dental caries. A total of 445 genes were identified with two fold or greater difference in the expression level. At least 85 genes were reported abundant in health and 360 more abundant in disease suggesting that this approach may contribute to improved future diagnosis and treatment. Another comprehensive study on human tooth pulp was done by 2D-gel electrophoresis followed by nano-liquid chromatography tandem mass spectrometry (LC/MS). This approach detected 342 proteins in total with a high confidence, and two proteins were distinguished in human samples [37]. Very recently, Eckhard *et al.* [96] attempted in depth dental pulp proteome with N-Terminome by the help of the terminal amine isotropic labelling of substrates (TAILS) approach and identified 17 missing protein candidates for the Chromosome-centric Human Proteome Project (C-HPP; www.c-hpp.og). Missing proteins can be defined as proteins that show only transcriptomic evidences and an expected sequence (or suggested by homology) or partly detected proteins. Furthermore, there are transcript evidences for the survival of the corresponding proteins available without conclusive mass spectrometry data [97].

Periodontal ligament (PDL) is another fibrous connective tissue containing heterogeneous cell population and type 1collagen fibers abundantly. They play a key role in maintaining PDL space, homeostasis, and anchorage, as well as maintaining and providing regeneration or repair of periodontium in response to disease and mechanical trauma. Only a few studies reported on PDL cellular components at genomics and proteomics level but it is very essential to understand the unique features and functions. Reichenberg *et al.* [38] reported a first study on periodontal ligament (PDL) fibroblast proteome for understanding physiology and regulation of PDL and identifying disease related protein markers. In this study 900 spots were detected and 117 proteins spot identified with 74 different genes. In another study on exploring the early osteogenic differential protein-profile in human PDL cells [98], 29 differentially expressed proteins during osteogenic differentiations were reported [98] which have been primarily linked to the cell membrane-binding, cytoskeleton, nuclear regulations, matrix synthesis signal conduction and metabolic enzymes [99]. Proteomics may shed light on these complex functional details of these intra- and inter-cellular processes.

## 5. Dental Materials Proteomics

Concurrent application of genomics and proteomics have revolutionized dentistry by allowing the identification and characterization of oral tissues (soft, hard, and liquid), and also help in understanding them on the molecular level [10]. By definition, dental materials are those materials or devices which interact with the oral environment in physio-chemical, mechanical, and biological aspects [100,101,102,103,104,105,106]. Hence, dental materials should be biocompatible and interact without causing any toxicity. Many approaches have been used previously to analyze the success of dental materials and failure at a cellular level. Very recently, Ryta *et al.* [107] studied elution of unreacted triethylene glycol dimethacrylate (TEGDMA) from Smart Dentine Replacement (SDR™), Dentsply International, UK bulk-fill dental composite by using HPLC. In this study, polymerized specimens were treated with four solutions (100% ethanol, 75% ethanol, distilled water, and 100% methanol) with different concentration to evaluate direct dental pulp toxicity of unreacted TEGDMA monomer. It was confirmed through HPLC that the toxicity of unreacted TEGDMA towards dental pulp established during the first hour after the placement of resin. Dental adhesive systems were analyzed by a research group for the quantification of monomer elution and carbon–carbon double bonds in dental adhesive system using reverse-phase HPLC, and observed that no correlation exists between the resin dentin bonding of adhesives and the elution of unreacted monomers [108]. However, further proteomic analysis of materials on the molecular level is needed to understand the changes in proteomes of failed or successful implants. Some of the studies reported in the last decade on proteomics of dental materials are listed in Table 3.

## 6. Conclusions

With the help of “omics” (genomics, transcriptomics, proteomics, metabolomics, and metagenomics) many hidden compositions, behavior and metabolisms of dental tissues and oral fluids have been analyzed in the last fifteen years. These scientific disciplines helped the gathering of valuable information of the human proteome and will complete the Human Proteome Project (HPP) [117]. Proteomics tools have provided remarkable information regarding dental tissues and oral fluids [118]. The overall analysis on proteomics in dentistry shows that more studies directed toward structural formation, diagnosis, and pathogenesis but very limited studies on evaluation of treatment, prevention of diseases, and prognosis of interventions. To sum up, all proteomic tools can help to fill the gaps of the unexplored aspects of oral health and dental sciences.

## Figures and Tables

**Table 1:** Detailed discussion of oral diseases protein analysis using proteomic tools.

Sample	Disease Condition	Proteomic Tools	Identified Markers	References
Whole mouth saliva (WS)	Oral squamous cell carcinoma	Shotgun proteome analysis, Western blotting (WB) and Enzyme Linked Immuno-Sorbent Assay (ELISA)	MRP14, M2BP, CD59, catalase, profilin, M2BP, involucrin, histone H1, S100A12, and S100P	[17]
WS	Denture stomatitis	Surface-Enhanced Laser Desorption/Ionization (SELDI) time-of-flight-(TOF)/ mass spectrometry (MS), liquid chromatography (LC)- Matrix-Assisted Laser Desorption/Ionization (MALDI)-TOF-MS	Statherin, desmocollin-2, kininogen-1, carbonic anhydrase-6, cystatin SN, cystatin c, peptidyl-prolyl cis-trans isomerase and immunoglobulin fragments	[18]
WS	Primary Sjögren’s syndrome	two dimensional electrophoresis (2-DE), MALDI-TOF/MS, WB and ELISA	Carbonic anhydrase VI, α-amylases precursor, epidermal fatty acid binding protein (E-FABP), macroglobulin (b-2), immunoglobulin k light chain (IGK-light chain) and glyceraldehydes-3-phosphate dehydrogenase (G3PDH)	[19]
WS	Secondary Sjögren’s syndrome	2-DE, MALDI-TOF-MS, WB and ELISA	Decrease ↓ Proline rich proteins (PRPs), ↓ Cystatin C, ↓ Lysozyme C and histatin, Increase ↑ Kallikrein and defensins	[20]
WS	GVHD	Tandem MS & ELISA	IL-1 receptor antagonist and cystatin B	[21]
WS	Protein-energy undernutrition	2-DE Gel and Image Master two dimensional 2D	Cyclic-dependent protein kinase	[22]
WS	Squamous cell carcinoma (head and neck region)	C4 Reverse Phase-High Pressure Liquid Chromatography (RP-HPLC), and LC-MS/MS	MRP14, Profilin, CD59, catalase and M2BP	[23]
WS	Diabetes (type-2)	2D-LC-MS/MS, WB and ELISA	α-1-antitrypsin (A1AT), α-2 macroglobulin (A2MG), transthyretin (TTR), salivary α -amylase (AMYS), cystatin C (Cys-C)	[24]
WS	Edentulous patient with type-2 diabetes	2D-LC-MS/MS	Serum amyloid-A and glyceraldehyde-3-phosphate dehydrogenase are increased, serotransferrin and amylase, palate, lung and nasal epithelium associated proteins (PLUNC) are reduced	[25]
Unstimulated whole mouth saliva (USWS)	Squamous cell carcinoma (oral mucosa)	Ultra-Pressure Liquid Chromatography (UPLC-MS), Hydrophilic Interaction Liquid Chromatography (HILIC)	l-carnitine, choline, betaine and pipecolinic acid	[26]
Saliva (parotid glands)	Caries	HPLC-MS/MS	matrix metalloproteinase-9 (MMP9), mucin-7 (MUC7), lactotransferrin (LTF), carbonic anhydrase 6 (CA6), azurocidin (AZU), and cold agglutinin	[27]
WS	Orthodontic tooth movement	2-DE, MALDI-TOF/tandem mass spectrometry (TEM)	Protein S100-A9, CRISP-3, Immunoglobulin J chain and Ig α-1 chain C region	[28]
USWS	Aggressive periodontitis	2-DE/HPLC–Electrospray Ionization (ESI)-MS	Increase in serum albumin, immunoglobulin Ig γ2, α2 chain C region, zinc-α2 glycoprotein, salivary α-amylase and vitamin D-binding proteins. Decrease in lactotransferrin, carbonic anhydrase 6, elongation factor 2, 14-3-3 sigma, short palate, lung and nasal epithelium carcinoma-associated protein-2	[29]
USWS	Periodontitis chronic	2-DE/MALDI-TOF/TOF MS and nLC-Q-TOF	Rise in serum albumin, hemoglobin, immunoglobulin and α-amylase	[30]
WS	Periodontitis in obese patient	SELDI-TOF-MS	Albumin, haemoglobin (α and β chains) and α-defensins (1, 2 & 3)	[31]
USWS	Gingivitis	2-DE/MALDI-TOF/TOF MS and nLC-Q-TOF	Zymogen granule protein-16 homolog B mucin, S100-A9, histatin, proline-rich-protein, 3, lipocalin-1 precursor, carbonic anhydrase 6, prolactin-induced protein, cystatin, keratins	[32]
Dental cementum	-	Nano-Acuity HPLC and LTQ-FT ultra	Osteomodulin (OMD), biglycan (BGN), insulin-like growth factor II (IGF2), pigment epithelium-derived factor (SERPINF1) and POSTN	[33]
Fine Needle Aspiration (FNA) fluid	Parotid gland tumor (Benign origin)	Nano LC-ESI-MS/MS and LTQ-Qrbitrap velos analysis and Western blot analysis	Ig γ-1 and kappa chain and Ig α-1 chain C regions, S100A9, macrophage capping proteins, apolipoprotein E and α crystalline B chain, annexin (A1 and A4)	[34]
Gingival crevicular fluids (GCF)	Gingivitis and chronic periodontitis	2-DE-LC-ESI-MS and Nano-LC-ESI-MS	Fibronectin, keratin, neutrophil, defensin3, Immunoglobulins, lactotransferrin precursor, 14-3-3 protein ζ/δ and α-actinin	[35]
Dentin	-	LC-MS/MS	Biglycan, osteoglycin, osteopontin, osteocalcin, asporin, lumican, mimecan, DSPP and SOD3	[36]
Dental pulp	-	2-DE, Nano-LCMS/MS	342 proteins identified	[37]
Periodontal fibers (PDL)	-	2-DE, MALDI-TOF, Western blot,	117 proteins identified	[38]
Acquired enamel pellicle (AEP)	-	LC-ESI-MS/MS	130 proteins identified	[39]

**Table 2:** Profiling and proteomic tools used for the detection and characterization of gingival crevicular fluid (GCF) proteins.

Author	Sample Collection Sites	Collection Method	Proteomic Tool	Number of Identified Proteins	Outcome of Study	Reference
Baliban *et al.*	Collected from pre-selected sites with probing depth >6 mm and <8 mm in periodontitis patients and for periodontaly health from mesio-buccal sites of first molar	Filter strips (Periopapers^®^, Interstate Drug. Exchange, Amityville, NY, USA)	Protein digest with trypsin, HPLC, fragmented analysis with tandem mass spectrometry (MS/MS)	432 human proteins identified (120 new)	Study identified novel biomarkers from GCF of periodontaly healthy and chronic periodontitis patients	[68]
Tsuchida *et al.*	Labial side of maxillary incisors without crown and restoration	Absorbent paper points (ZIPPERER^®^, Munich, Germany)	2-DE, Sodium-Dodecyl-Sulfate-Polyacrylamide Gel Electrophoresis (SDS-PAGE), WB analysis, HPLC with LTQ-XL, HPLC with LTQ-Orbitrap XL, LC-MS/MS	327 proteins identified	SOD1 and DCD were significantly increase ↑ in GCF of periodontal patients	[64]
Carneiro *et al.*	Healthy gingival sulcus of the second and third molar teeth	Periopapers^®^, USA	Trypsin digested followed by nano-flow liquid chromatography electrospray ionization tandem mass spectrometry (LC-ESI-MS/MS) analysis and ELISA for human albumin analysis	199 proteins identified	Provide proteins analysis of healthy periodontium and explore GCF composition with new groups of proteins specific to GCF microenvironment	[71]
Ngo *et al.*	Five deepest sites and molar sites except mesial surface were excluded	Microcaps (glass micocapillary tubes); Drummed Scientific, Brookmall, PA, USA	Matrix-assisted laser desposition/ionization time-of-flight (MALDI-TOF) mass spectrometry (MS)		GCF mass spectra could be best for analyzing attachment loss and diagnosis of periodontal diseases	[69]
Carina, *et al.*	Chronic Periodontitis patients sample were taken from different sites (5 deep sites, 5 shallow sites with gingivitis, and 4 without bleeding on probing sites)	Periopaper strip (ProFlow Inc., Amityville, NY, USA)	Reversed- phase (RP) LC-ESi-MS/MS and ELISA	230 proteins identified	Concluded marked differences in GCF proteomics in different disease profiles	[70]
Carneiro *et al.*	The pre-selected specific sites with moderate and severe chronic periodontal disease were defined by pocket depth of 5–7 mm (24 patients) and >7 mm (16 patients)	Periopaper strips (Oraflow, Plainview, NY, USA)	SDS-PAGE, Isotope-Coded-Affinity-Tag (ICAT) labelling, mTRAQ labelling, Nano-LC-ESI-MS/MS, Human Albumin ELISA Kit, and S100-A9 protein quantification by ELISA	238 proteins Identified	Innovative approach concluded the novel changes in host and microbial derived GCF proteome of periodontal patients	[72]
Rody Jr *et al.*	Collected from a deciduous second molar with radiographic evidence of root resorption on 1 quadrant (experimental site) and from the permanent first molar on the contralateral quadrant (control site) in the same jaw	Periopaper strips (Oraflow, Plainview, NY, USA)	One dimensional LC-MS and Two dimensional (2D) LC-MS	2789 proteins in control group and 2421 proteins in root resorption group	Mass spectrometry is useful tool for analyzing external root resorption	[73]
Kinney *et al.*	Collection from the mesio-buccal aspect of each site (tooth) for up-to 28 teeth per patient	Methylcellulose strip (Pro Flow, Inc., Amityville, NY, USA)	ELISA and Quantibody Human Cytokine Array (HCA)		This method offer improved patient monitoring and disease control	[74]
Huynh *et al.*	Collection were chosen based on how well they represented the healthy, gingivitis and chronic periodontitis inclusion criteria	Glass-microcapillary tube (Drummond Scientific, Brookmall, PA, USA)	One dimensional Gel-Electrophoresis and Nano-LC-ESI-MS	121 proteins identified	Concluded various biomarkers which differentiate between healthy periodontium, gingivitis and chronic periodontitis	[35]

**Table 3:** Use of proteomics techniques for dental materials analysis.

Author Name	Title of Study	Outcomes	References
Boyan *et al.*	Porcine fetal enamel matrix derivative enhances bone formation induced by demineralized freeze dried bone allograft *in vivo*	Emdogain contains a number of low-molecular-weight proteins (mainly amelogenins), associated with cementogenesis and osteogenesis during tooth development	[109]
Derhami *et al.*	Proteomic analysis of human skin fibroblasts grown on titanium: Novel approach to study molecular biocompatibility	Gain a better understanding of the molecular basis of biocompatibility of human skin fibroblast on titanium	[110]
Koin *et al.*	Analysis of the degradation of a model dental composite	Liquid chromatography mass spectrometry (LC-MS) found leaching of intact BisGMA and several degradation products that contained the bisphenol A moiety from the overlayer into distilled water after 2 weeks of aging	[111]
Jung *et al.*	Proteomic analysis in cyclosporin A (CsA)-induced overgrowth of human gingival fibroblast (HGF)	The CsA-treated HGF demonstrated that Prx 1 may play a crucial role in the HGF proliferation induced by CsA and proteomic analysis data provide an efficient approach in understanding the mechanisms of HGF proliferation by CsA	[112]
Taiyoji *et al.*	Identification of proteinaceous inhibitors of a cysteine proteinase (an Arg-specific gingipain) from Porphyromonas gingivalis in rice grain, using targeted-proteomics approaches	These results suggest that these rice proteins may be useful as nutraceutical ingredients for the prevention and management of periodontal diseases	[113]
Haigh *et al.*	Alterations in the salivary proteome associated with periodontitis	Results highlight the predominant involvement of S100 proteins in the host response during periodontitis	[113]
Zilm and Bartold *et al.*	Proteomic identification of proteinase inhibitors in the porcine enamel matrix derivative, EMD^®^	Enamel matrix derivatives (EMD) contains a number of high-molecular-weight compounds which include the proteinase inhibitors, fetuin A and α1-antichymotrypsin	[114]
Dorkhan *et al.*	Effects of saliva or serum coating on adherence of Streptococcus oralis strains to titanium	The adherence of LA11 and 89C strain to the moderately rough surfaces coated with saliva was more than twice that seen on the smooth saliva coated surfaces. This clearly demonstrates that surface topography is, at least to some degree, maintained in the presence of a saliva coating	[115]
Zhao *et al.*	Quantitative proteomic analysis of human osteoblast-like MG-63 cells in response to bio-inert implant material titanium and polyetheretherketone (PEEK)	Titanium and polyetheretherketone (PEEK) induces similar response in osteoblast proteome and PEEK causing worse proliferation was related to mRNA processing	[116]

## Abbreviations

BGLAP	Bone Gamma Carboxyglutamate Protein
cDNA	Complementary Deoxyribonucleic Acid
CHAD	Chondroadherin
C-HPP	Chromosome-Centric Human Proteome Project
CsA	Cyclosporin A
DNAse	Deoxyribonuclease
EMD	Enamel Matrix Derivative
ESI	Electrospray Ionization
FN	Fibronectin
FNA	Fine Needle Aspiration
HGF	Hepatocyte Growth Factor
GCF	Gingival Crevicular Fluid
GVHD	Graft Versus Host Disease
HA	Hydroxyapatite
HPP	Human Proteome Project
LC/MS	Liquid chromatography/mass spectrometry
LTQ-FT	Linear Ion Trap Mass Spectrometer
LUM	Lumican
mRNA	messenger Ribonucleic Acid
MS	Mass-Spectrometry
MALDI	Matrix-Assisted Laser Desorption/Ionization
PDL	Periodontal Ligament
PEEK	Polyetheretherketone
R-PCR	Realtime-Polymerase Chain Reaction
SDS-PAGE	Sodium dodecyl sulfate polyacrylamide gel electrophoresis
TAILS	Terminal Amine Isotropic Labelling of Substrates
TNN	Tenascin
USWS	Unstimulated Whole-Mouth Saliva
VIM	Vimentin
VTN	Vitronectin
WS	Whole-Mouth saliva
WB	Western Blotting
2-DE	2-Dimensional Electrophoresis

## References

[1] Bhattacharyya M. (2015). Protein structure and function: Looking through the network of side-chain interactions. Curr. Protein Pept. Sci..

[2] Alberts B. (1998). The cell as a collection of protein machines: Preparing the next generation of molecular biologists. Cell.

[3] Murzin A.G., Brenner S.E., Hubbard T., Chothia C. (1995). SCOP: A structural classification of proteins database for the investigation of sequences and structures. J. Mol. Biol..

[4] Luscombe N.M. (2001). Amino acid-base interactions: A three-dimensional analysis of protein-DNA interactions at an atomic level. Nucleic Acids Res..

[5] Nussinov R., Wolfson H.J. (1991). Efficient detection of three-dimensional structural motifs in biological macromolecules by computer vision techniques. Proc. Natl. Acad. Sci. USA.

[6] Wilkins M. (2009). Proteomics data mining. Exp. Rev. Proteom..

[7] Altelaar A.F.M., Munoz J., Heck A.J.R. (2013). Next-generation proteomics: Towards an integrative view of proteome dynamics. Nat. Rev. Genet..

[8] Latterich M., Abramovitz M., Leyland-Jones B. (2008). Proteomics: New technologies and clinical applications. Eur. J. Cancer.

[9] De Vries S., Bonvin A. (2008). How proteins get in touch: Interface prediction in the study of biomolecular complexes. Curr. Protein Pept. Sci..

[10] Chiappelli F., Covani U., Giacomelli L. (2010). Proteomics as it pertains to oral pathologies and dental research. Bioinformation.

[11] Hubbard M.J., Faught M.J., Carlisle B.H., Stockwell P.A. (2001). *ToothPrint*, a proteomic database for dental tissues. Proteomics.

[12] Jágr M., Eckhardt A., Pataridis S., Broukal Z., Dušková J., Mikšík I. (2014). Proteomics of human teeth and saliva. Physiol. Res..

[13] Rezende T.M.B., Lima S.M.F., Petriz B.A., Silva O.N., Freire M.S., Franco O.L. (2013). Dentistry proteomics: From laboratory development to clinical practice. J. Cell. Physiol..

[14] Amado F.M.L., Ferreira R.P., Vitorino R. (2013). One decade of salivary proteomics: Current approaches and outstanding challenges. Clin. Biochem..

[15] Cabras T., Iavarone F., Manconi B., Olianas A., Sanna M.T., Castagnola M., Messana I. (2014). Top-down analytical platforms for the characterization of the human salivary proteome. Bioanalysis.

[16] Seema S., Krishnan M., Harith A.K., Sahai K., Iyer S.R., Arora V., Tripathi R.P. (2014). Laser ionization mass spectrometry in oral squamous cell carcinoma. J. Oral Pathol. Med..

[17] Hu S., Arellano M., Boontheung P., Wang J., Zhou H., Jiang J., Elashoff D., Wei R., Loo J.A., Wong D.T. (2008). Salivary proteomics for oral cancer biomarker discovery. Clin. Cancer Res..

[18] Bencharit S., Altarawneh S.K., Baxter S.S., Carlson J., Ross G.F., Border M.B., Mack C.R., Byrd W.C., Dibble C.F., Barros S. (2012). Elucidating role of salivary proteins in denture stomatitis using a proteomic approach. Mol. BioSyst..

[19] Baldini C., Giusti L., Ciregia F., da Valle Y., Giacomelli C., Donadio E., Sernissi F., Bazzichi L., Giannaccini G., Bombardieri S. (2011). Proteomic analysis of saliva: A unique tool to distinguish primary Sjögren’s syndrome from secondary Sjögren’s syndrome and other sicca syndromes. Arthritis Res. Ther..

[20] Baldini C., Laura G., Laura B., Antonio L., Stefano B. (2008). Proteomic analysis of the saliva: A clue for understanding primary from secondary Sjögren’s syndrome?. Autoimmun. Rev..

[21] Devic I., Shi M., Schubert M.M., Lloid M., Izutsu K.T., Pan C., Missaghi M., Morton T.H., Mancl L.A., Zhang J. (2014). Proteomic analysis of saliva from patients with oral chronic graft-versus-host disease. Biol. Blood Marrow Transplant..

[22] Fonteles C.S.R., dos Santos C.F., da Silva Alves K.S., de Miranda Mota A.C., Damasceno J.X., Fonteles M.C. (2012). Comparative proteomic analysis of human whole saliva of children with protein-energy undernutrition. Nutrition.

[23] Dowling P., Robert W., Paula M., Michael H., Aongus C., Martin C. (2008). Analysis of the saliva proteome from patients with head and neck squamous cell carcinoma reveals differences in abundance levels of proteins associated with tumour progression and metastasis. J. Proteom..

[24] Rao P.V., Reddy A.P., Lu X., Dasari S., Krishnaprasad A., Biggs E., Roberts C.T., Nagalla S.R. (2009). Proteomic identification of salivary biomarkers of type-2 diabetes. J. Proteome Res..

[25] Border M.B., Schwartz S., Carlson J., Dibble C.F., Kohltfarber H., Offenbacher S., Buse J.B., Bencharit S. (2012). Exploring salivary proteomes in edentulous patients with type 2 diabetes. Mol. BioSyst..

[26] Wang Q., Gao P., Wang X., Duan Y. (2014). Investigation and identification of potential biomarkers in human saliva for the early diagnosis of oral squamous cell carcinoma. Clin. Chim. Acta.

[27] Yan G., Huang W., Xue H., Jia Y., Yang D. (2014). [Relationship between dental caries and salivary proteome by electrospray ionization ion-trap tandem mass spectrometry in children aged 6 to 8 years]. Hua Xi Kou Qiang Yi Xue Za Zhi.

[28] Ellias M.F., Zainal Ariffin S.H., Karsani S.A., Abdul Rahman M., Senafi S., Megat Abdul Wahab R. (2012). Proteomic analysis of saliva identifies potential biomarkers for orthodontic tooth movement. Sci. World J..

[29] Wu Y., Shu R., Luo L.-J., Ge L.-H., Xie Y.-F. (2009). Initial comparison of proteomic profiles of whole unstimulated saliva obtained from generalized aggressive periodontitis patients and healthy control subjects. J. Periodontal Res..

[30] Gonçalves L.D.R., Soares M.R., Nogueira F.C.S., Garcia C., Camisasca D.R., Domont G., Feitosa A.C.R., Pereira D.D.A., Zingali R.B., Alves G. (2010). Comparative proteomic analysis of whole saliva from chronic periodontitis patients. J. Proteom..

[31] Rangé H., Léger T., Huchon C., Ciangura C., Diallo D., Poitou C., Meilhac O., Bouchard P., Chaussain C. (2012). Salivary proteome modifications associated with periodontitis in obese patients. J. Clin. Periodontol..

[32] Gonçalves L.D.R., Soares M.R., Nogueira F.C.S., Garcia C.H.S., Camisasca D.R., Domont G., Feitosa A.C.R., Pereira D.A., Zingali R.B., Alves G. (2011). Analysis of the salivary proteome in gingivitis patients. J. Periodontal Res..

[33] Salmon C.R., Tomazela D.M., Ruiz K.G.S., Foster B.L., Paes Leme A.F., Sallum E.A., Somerman M.J., Nociti F.H. (2013). Proteomic analysis of human dental cementum and alveolar bone. J. Proteom..

[34] Donadio E., Giusti L., Seccia V., Ciregia F., da Valle Y., Dallan I., Ventroni T., Giannaccini G., Sellari-Franceschini S., Lucacchini A. (2013). New insight into benign tumours of major salivary glands by proteomic approach. PLoS ONE.

[35] Huynh A H.S., Veith P.D., McGregor N.R., Adams G.G., Chen D., Reynolds E.C., Ngo L.H., Darby I.B. (2015). Gingival crevicular fluid proteomes in health, gingivitis and chronic periodontitis. J. Periodontal Res..

[36] Park E.-S., Cho H.-S., Kwon T.-G., Jang S.-N., Lee S.-H., An C.-H., Shin H.-I., Kim J.-Y., Cho J.-Y. (2009). Proteomics analysis of human dentin reveals distinct protein expression profiles. J. Proteome Res..

[37] Eckhardt A., Jágr M., Pataridis S., Mikšík I. (2014). Proteomic analysis of human tooth pulp: Proteomics of human tooth. J. Endod..

[38] Reichenberg E., Redlich M., Cancemi P., Zaks B., Pitaru S., Fontana S., Pucci-Minafra I., Palmon A. (2005). Proteomic analysis of protein components in periodontal ligament fibroblasts. J. Periodontol..

[39] Siqueira W., Zhang W., Helmerhorst E.J., Gygi S., Oppenheim F.G. (2007). Identification of protein components in *in vivo* human acquired enamel pellicle using LC-ESI-MS/MS. J. Proteome Res..

[40] Khurshid Z., Naseem M., Sheikh Z., Najeeb S., Shahab S., Zafar M.S. (2015). Oral antimicrobial peptides: Types and role in the oral cavity. Saudi Pharm. J..

[41] He L.H., Swain M.V. (2008). Understanding the mechanical behaviour of human enamel from its structural and compositional characteristics. J. Mech. Behav. Biomed. Mater..

[42] Hubbard M.J., Kon J.C. (2002). Proteomic analysis of dental tissues. J. Chromatogr. B.

[43] Hubbard M.J., McHugh N.J., Mangum J.E. (2011). Exclusion of all three calbindins from a calcium-ferry role in rat enamel cells. Eur. J. Oral Sci..

[44] Hubbard M.J., McHugh N.J., Carne D.L. (2000). Isolation of ERp29, a novel endoplasmic reticulum protein, from rat enamel cells. Eur. J. Biochem..

[45] Hubbard M.J. (1996). Abundant calcium homeostasis machinery in rat dental enamel cells. Up-regulation of calcium store proteins during enamel mineralization implicates the endoplasmic reticulum in calcium transcytosis. Eur. J. Biochem..

[46] Hubbard M.J. (1995). Calbindin_28kDa_ and calmodulin are hyperabundant in rat dental enamel cells. Identification of the protein phosphatase calcineurin as a principal calmodulin target and of a secretion-related role for calbindin_28kDa_. Eur. J. Biochem..

[47] Moradian-Oldak J., Goldberg M. (2005). Amelogenin supra-molecular assembly *in vitro* compared with the architecture of the forming enamel matrix. Cells Tissues Organs.

[48] Bartlett J.D., Bernhard G., Michel G., Janet M.-O., Michael L.P., Malcolm L.S., Xin W., Shane N.W., Yan L.Z. (2006). Protein–protein interactions of the developing enamel matrix. Curr. Top. Dev. Biol..

[49] Dusevich V., Xu C., Wang Y., Walker M.P., Gorski J.P. (2012). Identification of a protein-containing enamel matrix layer which bridges with the dentine-enamel junction of adult human teeth. Arch. Oral Biol..

[50] Vymetal J., Slabý I., Spahr A., Vondrásek J., Lyngstadaas S.P. (2008). Bioinformatic analysis and molecular modelling of human ameloblastin suggest a two-domain intrinsically unstructured calcium-binding protein. Eur. J. Oral Sci..

[51] Wald T., Bednárová L., Osička R., Pachl P., Sulc M., Lyngstadaas S.P., Slaby I., Vondrášek J. (2011). Biophysical characterization of recombinant human ameloblastin. Eur. J. Oral Sci..

[52] Smith A.J., Scheven B.A., Takahashi Y., Ferracane J.L., Shelton R.M., Cooper P.R. (2012). Dentine as a bioactive extracellular matrix. Arch. Oral Biol..

[53] Petersson U., Hultenby K., Wendel M. (2003). Identification, distribution and expression of osteoadherin during tooth formation. Eur. J. Oral Sci..

[54] Jágr M., Eckhardt A., Pataridis S., Mikšík I. (2012). Comprehensive proteomic analysis of human dentin. Eur. J. Oral Sci..

[55] Robey P.G. (1996). Vertebrate mineralized matrix proteins: Structure and function. Connect. Tissue Res..

[56] Bosshardt D.D., Selvig K.A. (1997). Dental cementum: The dynamic tissue covering of the root. Periodontol. 2000.

[57] Hammarström L., Alatli I., Fong C.D. (1996). Origins of cementum. Oral Dis..

[58] Campisi G., Fede O.D., Roccia P., Nicola F.D., Falaschini S., Muzio L. (2006). Lo Saliva: Its value as a biological matrix and current methods of sampling. Eur. J. Inflamm..

[59] Bassim C.W., Ambatipudi K.S., Mays J.W., Edwards D.A., Swatkoski S., Fassil H., Baird K., Gucek M., Melvin J.E., Pavletic S.Z. (2012). Quantitative salivary proteomic differences in oral chronic graft-versus-host disease. J. Clin. Immunol..

[60] Chianeh Y.R., Prabhu K. (2014). Biochemical markers in saliva of patients with oral squamous cell carcinoma. Asian Pac. J. Trop. Dis..

[61] Levine M. (2011). Susceptibility to dental caries and the salivary proline-rich proteins. Int. J. Dent..

[62] Zhang L., Xiao H., Karlan S., Zhou H., Gross J., Elashoff D., Akin D., Yan X., Chia D., Karlan B. (2010). Discovery and preclinical validation of salivary transcriptomic and proteomic biomarkers for the non-invasive detection of breast cancer. PLoS ONE.

[63] Lamster I.B. (1997). Evaluation of components of gingival crevicular fluid as diagnostic tests. Ann. Periodontol..

[64] Tsuchida S., Satoh M., Umemura H., Sogawa K., Kawashima Y., Kado S., Sawai S., Nishimura M., Kodera Y., Matsushita K. (2012). Proteomic analysis of gingival crevicular fluid for discovery of novel periodontal disease markers. Proteomics.

[65] Tsuchida S., Satoh M., Sogawa K., Kawashima Y., Kado S., Ishige T., Beppu M., Sawai S., Nishimura M., Kodera Y. (2014). Application of proteomic technologies to discover and identify biomarkers for periodontal diseases in gingival crevicular fluid: A review. PROTEOM.-Clin. Appl..

[66] Kunimatsu K. (1990). Cathepsins B, H and L activities in gingival crevicular fluid from chronic adult periodontitis patients and exprimental gingivitis subjects. J. Periodontal Res..

[67] Dannies P.S. (1982). Protein degradation in health and disease. Ciba foundation symposium 75 (new series). Yale J. Biol. Med..

[68] Baliban R.C., Sakellari D., Li Z., DiMaggio P.A., Garcia B.A., Floudas C.A. (2012). Novel protein identification methods for biomarker discovery via a proteomic analysis of periodontally healthy and diseased gingival crevicular fluid samples. J. Clin. Periodontol..

[69] Ngo L.H., Darby I.B., Veith P.D., Locke A.G., Reynolds E.C. (2013). Mass spectrometric analysis of gingival crevicular fluid biomarkers can predict periodontal disease progression. J. Periodontal Res..

[70] Silva-Boghossian C.M., Colombo A.P.V., Tanaka M., Rayo C., Xiao Y., Siqueira W.L. (2013). Quantitative proteomic analysis of gingival crevicular fluid in different periodontal conditions. PLoS ONE.

[71] Carneiro L.G., Venuleo C., Oppenheim F.G., Salih E. (2012). Proteome data set of human gingival crevicular fluid from healthy periodontium sites by multidimensional protein separation and mass spectrometry. J. Periodontal Res..

[72] Carneiro L.G., Nouh H., Salih E. (2014). Quantitative gingival crevicular fluid proteome in health and periodontal disease using stable isotope chemistries and mass spectrometry. J. Clin. Periodontol..

[73] Rody W.J., Holliday L.S., McHugh K.P., Wallet S.M., Spicer V., Krokhin O. (2014). Mass spectrometry analysis of gingival crevicular fluid in the presence of external root resorption. Am. J. Orthod. Dentofac. Orthop..

[74] Kinney J.S., Morelli T., Oh M., Braun T.M., Ramseier C.A., Sugai J.V., Giannobile W.V. (2014). Crevicular fluid biomarkers and periodontal disease progression. J. Clin. Periodontol..

[75] Siqueira W.L., Custodio W., McDonald E.E. (2012). New insights into the composition and functions of the acquired enamel pellicle. J. Dent. Res..

[76] Vukosavljevic D., Custodio W., Buzalaf M.A.R., Hara A.T., Siqueira W.L. (2014). Acquired pellicle as a modulator for dental erosion. Arch. Oral Biol..

[77] Lee Y.H., Zimmerman J.N., Custodio W., Xiao Y., Basiri T., Hatibovic-Kofman S., Siqueira W.L. (2013). Proteomic evaluation of acquired enamel pellicle during *in vivo* formation. PLoS ONE.

[78] Lendenmann U., Grogan J., Oppenheim F.G. (2000). Saliva and dental pellicle—A review. Adv. Dent. Res..

[79] Reich M., Hannig C., Al-Ahmad A., Bolek R., Kummerer K. (2012). A comprehensive method for determination of fatty acids in the initial oral biofilm (pellicle). J. Lipid Res..

[80] Siqueira W.L., Helmerhorst E.J., Zhang W., Salih E., Oppenheim F.G. (2007). Acquired enamel pellicle and its potential role in oral diagnostics. Ann. N. Y. Acad. Sci..

[81] Mayhall C.W. (1970). Concerning the composition and source of the acquired enamel pellicle of human teeth. Arch. Oral Biol..

[82] Armstrong W.G. (1966). Amino-acid composition of the acquired pellicle of human tooth enamel. Nature.

[83] Siqueira W.L., Margolis H.C., Helmerhorst E.J., Mendes F.M., Oppenheim F.G. (2010). Evidence of intact histatins in the *in vivo* acquired enamel pellicle. J. Dent. Res..

[84] Zimmerman J.N., Custodio W., Hatibovic-Kofman S., Lee Y.H., Xiao Y., Siqueira W.L. (2013). Proteome and peptidome of human acquired enamel pellicle on deciduous teeth. Int. J. Mol. Sci..

[85] Yu T., Volponi A.A., Babb R., An Z., Sharpe P.T. (2015). Stem cells in tooth development, growth, repair, and regeneration. Curr. Top. Dev. Biol..

[86] Mjör I.A., Sveen O.B., Heyeraas K.J. (2001). Pulp-dentin biology in restorative dentistry. Part 1: Normal structure and physiology. Quintessence Int..

[87] Farges J.-C., Alliot-Licht B., Renard E., Ducret M., Gaudin A., Smith A.J., Cooper P.R. (2015). Dental pulp defence and repair mechanisms in dental caries. Mediat. Inflamm..

[88] Robertson A., Lundgren T., Andreasen J.O., Dietz W., Hoyer I., Norén J.G. (1997). Pulp calcifications in traumatized primary incisors. A morphological and inductive analysis study. Eur. J. Oral Sci..

[89] Yamazoe T., Aoki K., Simokawa H., Ohya K., Takagi Y. (2002). Gene expression of bone matrix proteins in a calcified tissue appeared in subcutaneously transplanted rat dental pulp. J. Med. Dent. Sci..

[90] Zafar M.S., Khurshid Z., Almas K. (2015). Oral tissue engineering progress and challenges. Tissue Eng. Regen. Med..

[91] Sandanayake N.S., Sinclair J., Andreola F., Chapman M.H., Xue A., Webster G.J., Clarkson A., Gill A., Norton I.D., Smith R.C. (2011). A combination of serum leucine-rich α-2-glycoprotein 1, CA19–9 and interleukin-6 differentiate biliary tract cancer from benign biliary strictures. Br. J. Cancer.

[92] Pääkkönen V., Ohlmeier S., Bergmann U., Larmas M., Salo T., Tjäderhane L. (2005). Analysis of gene and protein expression in healthy and carious tooth pulp with cDNA microarray and two-dimensional gel electrophoresis. Eur. J. Oral Sci..

[93] Wei X., Wu L., Ling J., Liu L., Liu S., Liu W., Li M., Xiao Y. (2008). Differentially expressed protein profile of human dental pulp cells in the early process of odontoblast-like differentiation *in vitro*. J. Endod..

[94] Tete S., Mastrangelo F., Scioletti A.P., Tranasi M., Raicu F., Paolantonio M., Stuppia L., Vinci R., Gherlone E., Ciampoli C. (2008). Microarray expression profiling of human dental pulp from single subject. Clin. Investig. Med..

[95] McLachlan J.L., Smith A.J., Bujalska I.J., Cooper P.R. (2005). Gene expression profiling of pulpal tissue reveals the molecular complexity of dental caries. Biochim. Biophys. Acta Mol. Basis Dis..

[96] Eckhard U., Marino G., Abbey S.R., Tharmarajah G., Matthew I., Overall C.M. (2015). The human dental pulp proteome and N-terminome: Levering the unexplored potential of semitryptic peptides enriched by TAILS to identify missing proteins in the human proteome project in underexplored tissues. J. Proteome Res..

[97] Paik Y.-K., Hancock W.S. (2012). Uniting ENCODE with genome-wide proteomics. Nat. Biotechnol..

[98] Wu L., Wei X., Ling J., Liu L., Liu S., Li M., Xiao Y. (2009). Early osteogenic differential protein profile detected by proteomic analysis in human periodontal ligament cells. J. Periodontal Res..

[99] Wu L., Wei X., Ling J., Liu L. (2009). A differential expression proteomic study of human periodontal ligament cell during osteogenic differentiation. Zhong Hua Kou Qiang Yi Xue Za Zhi.

[100] Khurshid Z., Zafar M., Qasim S., Shahab S., Naseem M., AbuReqaiba A. (2015). Advances in nanotechnology for restorative dentistry. Materials.

[101] Sheikh Z., Najeeb S., Khurshid Z., Verma V., Rashid H., Glogauer M. (2015). Biodegradable materials for bone repair and tissue engineering applications. Materials.

[102] Najeeb S., Zafar M.S., Khurshid Z., Siddiqui F. (2016). Applications of polyetheretherketone (PEEK) in oral implantology and prosthodontics. J. Prosthodont. Res..

[103] Matinlinna J.P., Zeeshan S., Mohamed-Nur A., Nader H., Mohammad A.J., Zohaib K. (2014). Barrier membranes for periodontal guided tissue regeneration applications. Handbook of Oral Biomaterials.

[104] Najeeb S., Khurshid Z., Matinlinna J.P., Siddiqui F., Nassani M.Z., Baroudi K. (2015). Nanomodified peek dental implants: Bioactive composites and surface modification—A review. Int. J. Dent..

[105] Najeeb S., Khurshid Z., Zafar M.S., Ajlal S. (2016). Applications of light amplification by stimulated emission of radiation (lasers) for restorative dentistry. Med. Princ. Pract..

[106] Naseem M., Khurshid Z., Khan H.A., Niazi F., Shahab S., Zafar M.S. (2015). Oral health challenges in pregnant women: Recommendations for dental care professionals. Saudi J. Dent. Res..

[107] Łagocka R., Jakubowska K., Chlubek D., Buczkowska-Radlińska J. (2015). Elution study of unreacted TEGDMA from bulk-fill composite (SDR™ Dentsply) using HPLC. Adv. Med. Sci..

[108] Miletic V., Santini A., Trkulja I. (2009). Quantification of monomer elution and carbon–carbon double bonds in dental adhesive systems using HPLC and micro-Raman spectroscopy. J. Dent..

[109] Boyan B.D., Weesner T.C., Lohmann C.H., Andreacchio D., Carnes D.L., Dean D.D., Cochran D.L., Schwartz Z. (2000). Porcine fetal enamel matrix derivative enhances bone formation induced by demineralized freeze dried bone allograft *in vivo*. J. Periodontol..

[110] Derhami K., Zheng J., Li L., Wolfaardt J.F., Scott P.G. (2001). Proteomic analysis of human skin fibroblasts grown on titanium: Novel approach to study molecular biocompatibility. J. Biomed. Mater. Res..

[111] Koin P.J., Kilislioglu A., Zhou M., Drummond J.L., Hanley L. (2008). Analysis of the degradation of a model dental composite. J. Dent. Res..

[112] Jung J.Y., Kang G.C., Jeong Y.J., Kim S.H., Kwak Y.G., Kim W.J. (2009). Proteomic analysis in cyclosporin a-induced overgrowth of human gingival fibroblasts. Biol. Pharm. Bull..

[113] Taiyoji M., Shitomi Y., Taniguchi M., Saitoh E., Ohtsubo S. (2009). Identification of proteinaceous inhibitors of a cysteine proteinase (an Arg-specific gingipain) from Porphyromonas gingivalis in rice grain, using targeted-proteomics approaches. J. Proteome Res..

[114] Zilm P.S., Bartold P.M. (2011). Proteomic identification of proteinase inhibitors in the porcine enamel matrix derivative, EMD^®^. J. Periodontal Res..

[115] Dorkhan M., Chávez de Paz L.E., Skepö M., Svensäter G., Davies J.R. (2012). Effects of saliva or serum coating on adherence of Streptococcus oralis strains to titanium. Microbiology.

[116] Zhao M., An M., Wang Q., Liu X., Lai W., Zhao X., Wei S., Ji J. (2012). Quantitative proteomic analysis of human osteoblast-like MG-63 cells in response to bioinert implant material titanium and polyetheretherketone. J. Proteom..

[117] Oakley M., Barthel D., Bykov Y., Garibaldi J., Burke E., Krasnogor N., Hirst J. (2008). Search strategies in structural bioinformatics. Curr. Protein Pept. Sci..

[118] Khurshid Z., Shariq N., Maria M., Syed F.M., Syed Q.R., Sana Z., Farshid S., Muhammad S.Z. (2016). Histatin peptides: Pharmacological functions and its applications in dentistry. Saudi Pharm. J..

